# The human ATF1 rs11169571 polymorphism associated with risk of nasopharyngeal carcinoma in Southern Chinese populations

**DOI:** 10.1002/cam4.2022

**Published:** 2019-03-23

**Authors:** Shutang Peng, Guo‐Liang Huang, Nansong Xu, Yan Lu, Liuyan Zeng, Xin Li, Shengqun Luo, Xiaoming Lyu, Qiang Jiang, Tong Li, Zhiwei He

**Affiliations:** ^1^ China‐American Cancer Research Institute Dongguan Scientific Research Center Guangdong Medical University Dongguan China; ^2^ Key Laboratory for Epigenetics of Dongguan City Dongguan China; ^3^ Key Laboratory for Medical Molecular Diagnostics of Guangdong Province Dongguan China; ^4^ Department of Health Management Center The Affiliated Hospital of Guangdong Medical University Zhanjiang Guangdong China; ^5^ Cancer Research Institute Southern Medical University Guangzhou China; ^6^ Department of Laboratory Medicine The Third Affiliated Hospital of Southern Medical University Guangzhou China

**Keywords:** ATF1, microRNA, nasopharyngeal carcinoma, polymorphism, risk

## Abstract

Our previous work reported activating transcription factor 1 (ATF1) is a promotive factor of nasopharyngeal carcinoma (NPC) tumorigenesis. This study is to further explore the association between the human ATF1 rs11169571 polymorphism and the risk of NPC occurrence. The association between ATF1 rs11169571 and risk of NPC occurrence was investigated in clinical samples of 560 patients and 661 controls obtained from southern China with high incidence of NPC. The genotypes were detected by PCR‐RFLP. The differential expression activity of alleles ‐T and ‐C was analyzed with CNE‐2 and C666‐1 cells by luciferase reporter assay. Our data suggested that the allelic frequency and genotypes were significantly different between patients and controls. Compared to the TT homozygote, the TC and CC genotypes have been shown to be significantly decreased in NPC patients (OR = 0.494, 95% CI = 0.387‐0.629, *P* < 0.001 and OR = 0.556, 95% CI = 0.364‐0.851, *P* = 0.007, respectively). Compared to the ‐T allele, the ‐C allele is a factor of decreased risk in NPC (OR = 0.642, 95% CI = 0.537‐0.767, *P* < 0.001). Luciferase reporter activity revealed that the ‐T allele confers a higher expression activity than the ‐C allele in CNE2 cells and C666‐1 cells. In conclusion, ATF1 rs11169571 which could affect the expression of ATF1 is associated with NPC risk.

## 1 INTRODUCTION

Nasopharyngeal carcinoma (NPC) possesses a high incidence in Southeast Asia as well as Southern China.^1^, ^2^, ^3^ Significant ethnic susceptibility of NPC and familial clustering indicates that changes in inherited genes play an important role in NPC.^4^, ^5^, ^6^ It is very important to identify the genes closely related to the risk and development of NPC.

Activating transcription factor 1 (ATF1), which is a leucine‐zipper (bZIP) transcription factor of the ATF/CREB family, plays a key role within tumor progression.^7^, ^8^ ATF1 is first thought to be associated with tumors as a fusion gene that promoted clear cell sarcoma's cancer development.^9^ ATF1 is involved in the transcription regulation of FRA‐1, highly expressed in multiple tumors.^10^, ^11^ In addition, activation of ATF1 plays a significant role in the survival of gastric cancer cells by upregulation of Bcl‐2.^12^Date has shown that ATF1 enhanced Bcl‐2 expression in the promotion of NPC tumorigenesis, in our previous study.^13^

NPC has been regarded as an Epstein‐Barr virus (EBV)‐associated epithelial malignancy.^14^, ^15^ LMP1, a latent membrane protein coded by EBV genes, has been confirmed as the only malignant transformation gene of EBV.^16^, ^17^ LMP1 seems to be induced by proteins of CREB/ATF.^18^, ^19^, ^20^, ^21^ EBV gene expression is initiated by the activation of the BamHI W promoter (Wp), which has been activated by ATF/CREB transcription factors.^22^ With those evidences, we speculated that ATF1 might be related to the NPC's risk.

Recently, the SNP rs11169571 located in 3′‐UTRs of was found to modify miRNA binding and be associated with the risk of hypertension.^23^ We infer that this SNP may also be related to the cancer's development. Our study aims to investigate the association between ATF1 rs11169571 and nasopharyngeal carcinoma's risk.

## 2 MATERIALS AND METHODS

### 2.1 Study population

The population on the basis of case‐control research was implemented for assessing the association of ATF1 polymorphism with the susceptibility of NPC. The investigated population in Guangdong area in the study including 560 patients with nasopharyngeal carcinoma (NPC) and 661 healthy controls had been recruited from Affiliated Hospital of Guangdong Medical University (313 patients and 661 healthy controls) and Nanfang hospital, Southern Medical University (247 patients), from July 2010 to October 2015. The mean ages of normal controls as well as NPC patients were 47.38 ± 11.67 and 49.38 ± 15.60, respectively.

All of the patients were diagnosed through histopathology evidence and did not gain any treatment before the blood drawing. The clinicopathological data containing distant metastasis, nodal status as well as tumor size were clinically determined by magnetic resonance or computed tomography (CT) scan. The study protocol was approved by the Ethics Committees of Guangdong Medical University.

### 2.2 Genotyping detection

**Figure 1:**
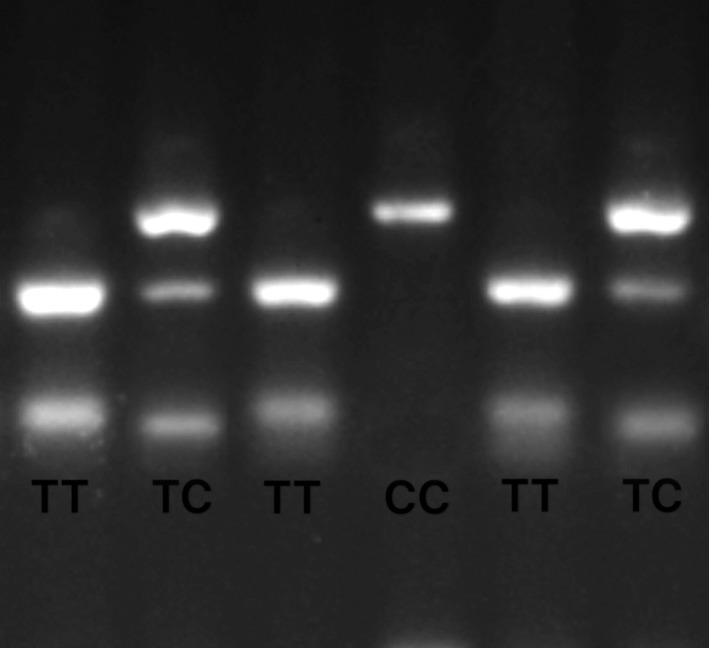
PCR‐RLFP detection results for ATF1 rs11169571 genotyping

The blood samples collected in anticoagulant tube were stored at −80°C. Genomic DNA was prepared from the overall blood samples by adopting a TIANAMP Blood DNA kit (Tiangen Biotech, Beijing, China). Polymerase chain reaction restriction fragment length polymorphism (PCR‐RFLP) was adopted for detecting the genotypes. The primers were synthesized by Sangon Biotech (Shanghai). The primers adopting for the amplification of PCR could be seen below: forward, 5′‐CGCACGATATCTAGTGACAGAGG‐3′; reverse, 5′‐TGCAAACCTGTAGGGTAAATGG‐3′. The PCR products were digested with the FastDigest HindIII enzymes (Thermo Fisher Scientific, USA), at 37°C for more than 4 hours or overnight, to identify the respective genotypes. After that, the genotype was recognized by 2.5% agarose gel electrophoresis for the digestion of the goods, with three bands implied TC genotype, two bands implied TT as well as one band implied CC (Figure 1). For verifying the accuracy, 5% of all samples was selected randomly and delivered to Sangon Biotech for sequencing validation of the PCR‐RFLP results.

### 2.3 Construction of reporter plasmids

Two wild‐type (‐T) or mutant‐type (‐C), pGL3 firefly luciferase reporter plasmids, including ATF1's 3′‐UTR, were constructed by Generay Biotech after the luciferase (Shanghai, China). The sequences of 3′‐UTR region of ATF1 were cloned according to a previous study.^23^

### 2.4 Transient transfection and Luciferase reporter assays

Human nasopharyngeal carcinoma cells (CNE2 and C666‐1) were cultured with 10% fetal bovine serum within RPMI Medium 1640 and were seeded into 24 ‐well culture plated with 4 x10^4^ cells per well overnight. Meanwhile, 1000 ng of wild‐type (‐T) and 10 ng PRL‐TK vector (Renilla luciferase plasmid) (or mutant‐type (‐C) ATF1) reporter construct was co‐transfected by adopting the transfection reagent of DNA based on jetPEI^™^ polymer (Polypus‐Transfection Inc, New York, America) in accordance with the recommendations of the manufacturer. Twelve hours after plating, the medium was changed. The cells were harvested 36 hours after transfection as well as discussion by adopting the Dual‐Luciferase Reporter Assay System (Promega, America), with Firefly signal to be Renilla signal as well as reporter gene to be the inner reference. The transfections were implemented for at least three times, with samples in triplicate by adopting various kinds of plasmid preparations for all the transfections.

### 2.5 Statistical analysis

Statistical analysis was carried out using SPSS 16.0 software and *P* < 0.05 was considered significant. The chi‐square analysis was adopted for comparing ATF1's allelic/genotypic frequencies and the presence of Hardy‐Weinberg equilibrium. The associations between the nasopharyngeal carcinoma and the ATF1 genotypes were assessed through the calculation of 95% of confidence interval (CI) as well as the odds ratio (OR), adopting the multivariate logistic regression analysis adjusted by gender as well as age.

## 3 RESULTS

### 3.1 Population characteristics

The demographic features as well as the risk factors of the healthy controls and enrolled patients are summarized in Table 1. Five hundred and sixty patients with NPC and 661 healthy controls from Guangdong province in the southern areas of our country entered the final analysis. No significant deviations between cases and controls were observed in the allocation of age (*P* = 0.289, Table 1) and sex (*P* = 0.112, Table 1).

**Table 1:** Characteristics of nasopharyngeal carcinoma patients and controls

Characteristics	Patient	Control	*P* value
Age mean	47.38 ± 11.67	49.38 ± 15.60	0.159
Age			
<45	233 (41.6%)	245 (37.1%)	0.295
≥45	327 (58.4%)	416 (62.9%)	
Gender			
Male	321 (57.3%)	359 (54.3%)	0.112
Female	239 (42.7%)	302 (45.7%)	
Primary tumor extension			
T1 + T2	52 (36.6%)	__	__
T3 + T4	90 (43.4%)		
Lymph node status			
N0	11 (7.7%)	__	__
N1 + N2 + N3	131 (92.3%)		
Metastasis			
No	123 (86.6%)	__	__
Yes	19 (13.4%)		

### 3.2 Association between genotype and allele distribution of ATF1 rs11169571 and risk of nasopharyngeal carcinoma

The allele as well as genotype distributions of the ATF1 rs11169571 SNPs, in the cases and controls, is summarized in Table 2. The minor allele frequencies of rs11169571 in case and control were 24.4 and 33.4%, respectively, whereas the MAF was 34.1% in the 1000 Genomes project. The genotype frequencies of ATF1 rs11169571 polymorphism have been analyzed in all control subjects and it is shown to be in accordance with the Hardy‐Weinberg equilibrium (*P* = 0.057).

**Table 2:** Genotype and allele distribution of ATF1 rs11169571 in patients and controls

Polymorphism	Patient n (%)	Control n (%)	*P* value	OR (95% CI)
Genotype				
TT	330 (59%)	282 (42.7%)	< 0.001	
TC	187 (33.3%)	316 (47.8%)		
CC	43 (7.7%)	63 (9.5%)		
CC vs TT			0.007	0.556^a^ (0.364‐0.851)
TC vs TT			<0.001	0.494^a^ (0.387‐0.629)
CC + TC vs TT			<0.001	0.506^a^ (0.402‐0.637)
Allele				
T	847 (75.6%)	880 (66.6%)	<0.001	0.642 (0.537‐0.767)
C	273 (24.4%)	442 (33.4%)		

a Data were calculated by unconditional logistic regression with adjustment for age and gender.

In Table 2, significant difference was observed in three genotype frequencies (TT, TC, CC) of ATF1 rs11169571 between controls and patient groups (*P* < 0.001, Table 2), by chi‐square analysis. By comparing with the TT genotype, the TC heterozygote and CC homozygote both revealed a greatly reduced risk of NPC (OR = 0.494, 95% CI = 0.387‐0.629, *P* < 0.001 for TC heterozygote; and OR =0.556, 95% CI = 0.364‐0.851, *P* = 0.007 for CC homozygote, respectively, Table 2). Compared to the ‐T allele, the ‐C allele was associated with a decreased risk of NPC (OR = 0.642, 95% CI = 0.537‐0.767, *P* < 0.001, Table 2).

A total of 142 cases having complete clinical data are summarized in Table 3 to show the connection between clinicopathological parameters and genotype. There are no significant associations between genotypes in the SNP and the clinicopathological parameters, which includes lymph node status, original tumor extension, gender, age as well as metastasis in patients.

**Table 3:** Association of genotype and clinicopathological parameters in NPC patients^a^

	Genotype			
Characteristics	TT	TC	CC	*P*
Age				
<45	39 (60%)	21 (32%)	5 (8%)	0.368
≥45	39 (50.6%)	27 (35.1%)	11 (14.3%)	
Gender				
Male	55 (52.4%)	36 (34.2%)	14 (13.4%)	0.362
Female	23 (62.2%)	12 (34.4%)	2 (5.4%)	
Primary tumor extension				
T1 + T2	28 (53.9%)	17 (32.7%)	7 (13.4%)	0.819
T3 + T4	50 (55.6%)	31 (34.4%)	9 (10%)	
Lymph node status				
N0	9 (81.8%)	1 (9.1%)	1 (9.1%)	0.151
N1 + N2 + N3	69 (52.6%)	47 (35.9%)	15 (11.5%)	
Metastasis				
No	65 (52.9%)	43 (34.9%)	15 (12.2%)	0.408
Yes	13 (68.4%)	5 (26.4%)	1 (5.2%)	

a Data available only in 142 cases.

### 3.3 3′‐UTR activity of ATF1 rs11169571

**Figure 2:**
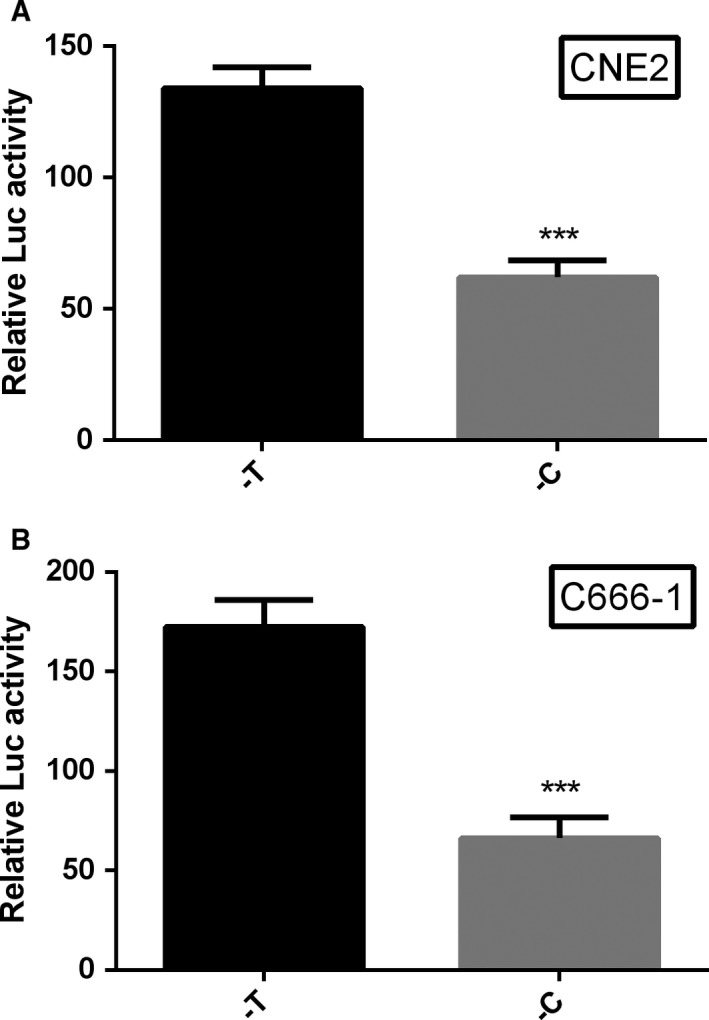
Luciferase activity of reporter gene expression driven by two alleles of ATF1 rs11169571, ***P < 0.001, compared to –T allele. A, in CNE2 cells; B, in C666‐1 cells

To verify whether the ATF1 rs11169571 affects its expression in the NPC cell, the luciferase reporter vector of the ATF1 gene's 3′‐UTR with either ‐C or ‐T allele of rs11169571 was cloned and co‐transfected into CNE2 and C666‐1 cells. The result revealed that the –C allele conferred a lower expression activity than that of the ‐T allele in NPC cells (*P* < 0.001, Figure 2).

## 4 DISCUSSION

ATF1 plays an important role in cancer progression and was found to promote nasopharyngeal carcinoma (NPC) tumorigenesis in our previous study. However, few studies analyzed the genetic variants in ATF1 and risk of cancer. To the best of our knowledge, this is the first study to report on the association between ATF1 polymorphism and risk of NPC occurrence.

The polymorphic site, ATF1 rs11169571, was analyzed in a group of ovarian/breast cancer patients with BRCA1 or BRCA2 mutation. It was reported that the heterozygote SNP carriers possessed a twofold increased risk to develop ovarian/breast cancer among BRCA2 mutation carriers on the basis of Kaplan‐Meier analyses as well as Cox regression at age of diagnosis.^24^ A case‐control study, including approximately 435 women patients who have breast cancer as well as 439 gender‐ and age‐matched tumor‐free individuals, implied that no significant association between the risk of breast cancer and rs11169571.^25^ Our results suggested that when compared to TT homozygote, both the CT heterozygote and CC homozygote were associated with a decreased risk of NPC. The ‐C allele was shown to be associated with a decreased risk of NPC. Our study which collected a large number of samples with NPC in high incidence area of Southern China would indicate a significant association between ATF1 and NPC risk.

Our previous study indicates that ATF1 plays a pro‐cancer role in NPC. By using luciferase reporter assays, it was significantly found that the fluorescence value of ‐T allele is higher than ‐C allele in CNE2 cell line and C666‐1 cell line. The result suggested that the ‐T allele carrier would have a higher expression of ATF1 than the ‐C allele carrier. These results which are consistent with our case‐control study become a reliable proof to prove that ‐C allele had been linked to NPC's reduced risk compared to ‐T allele.

As previously mentioned, ATF1 is able to facilitate Epstein‐Barr virus infection by enhancing the transcription of LMP1. However, there are no studies investigating the relationship between SNP of ATF1 and EBV infection. The genotype frequency of SNP rs2274084 on GJB2 gene was significantly different between EBV‐positive NPC and normal control.^26^ The C118T genotype on the excision repair cross‐complementing group 1 (ERCC1) gene was reported to be associated with plasma EBV DNA levels.^27^ In the present study, we found that rs11169571 on ATF1 was associated with the risk of NPC. However, we were unable to suggest an association between the SNP and the EBV status, which was not collected in this study. We speculated that the SNP on ATF1 could affect EBV status by modulating the expression of ATF1. It needs to be verified in the subsequent study.

3′‐UTRs influence the available pool of mRNAs which could be translated into proteins through the facilitation of the interactions with microRNA (miRNA) molecules. miRNAs are small‐sized noncoding RNAs which modulate the production of proteins through binding to short motifs considered to be miRNA‐binding sites, discovered mainly within the 3′‐UTR.^28^, ^29^, ^30^ SNPs in 3′‐UTR regions of the miRNA corresponding binding sites could impact miRNA expression and target mRNA translation.^31^ The rs11169571, located in the 3′‐UTR of ATF1, could affect hsa‐miR‐1283′s regulation to ATF1 and cause differences in the susceptibility to hypertension.^23^ Overexpression of miR‐1283 was suggested to inhibit the invasion as well as proliferation of cells through targeting ATF4 within glioma cells.^32^

Our data suggested that lack of correlation between ATF1 rs11169571 and clinicopathological parameters including age, gender, primary tumor extension, lymph node status, and metastasis in patients. However, lack of the correlation may be due to relatively limited number of cases with clinicopathological information.

All in all, our study suggested that ATF1 rs11169571 polymorphism was associated with nasopharyngeal cancer's risk in a Chinese population in Guangdong Province. The luciferase assay indicating a lower expression of ATF1 in the ‐C allele carrier was consistent with the association study. The ATF1 rs11169571 might be considered to be a potential biomarker for nasopharyngeal cancer risks. The mechanism of this SNP in NPC needed to be further studied.
